# Supplementary figures and images for: Impact of bread diet on intestinal dysbiosis and irritable bowel syndrome symptoms in quiescent ulcerative colitis: A pilot study
Source: PLoS One. 2024 Feb 16;19(2):e0297836. doi: 10.1371/journal.pone.0297836 (PMC10871487; doi:10.1371/journal.pone.0297836)

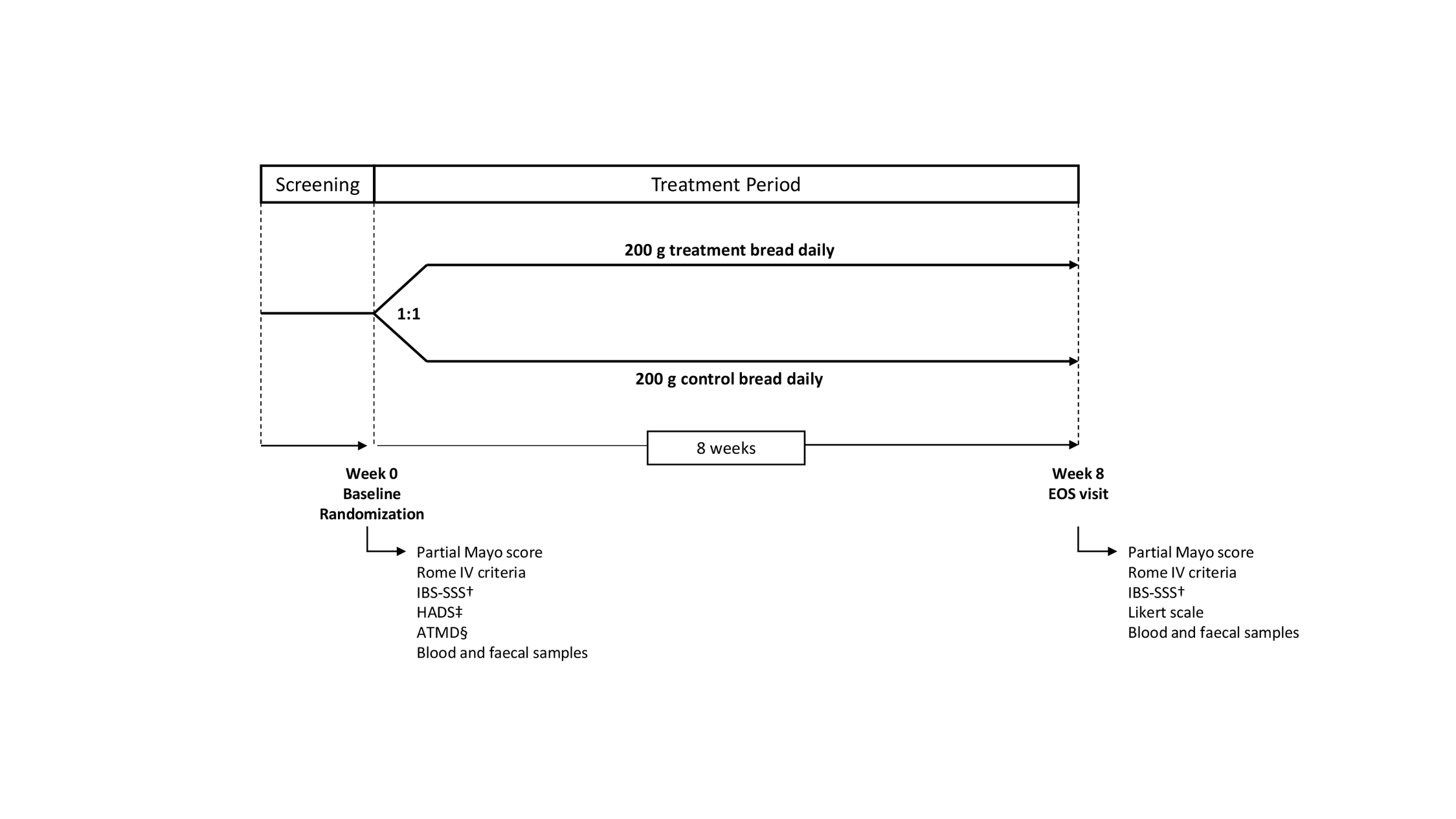

Supplement: S1 Fig — Intervention arms, baseline and end of study (EOS) visits, and reported outcome measures assessed at each time point. †IBS‐SSS, IBS-Symptom Severity Score, ‡HADS, Hospital Anxiety and Depression Scale, §ATMD, Adherence to Mediterranean Diet questionnaire. (TIF) [file pone.0297836.s005.tif]

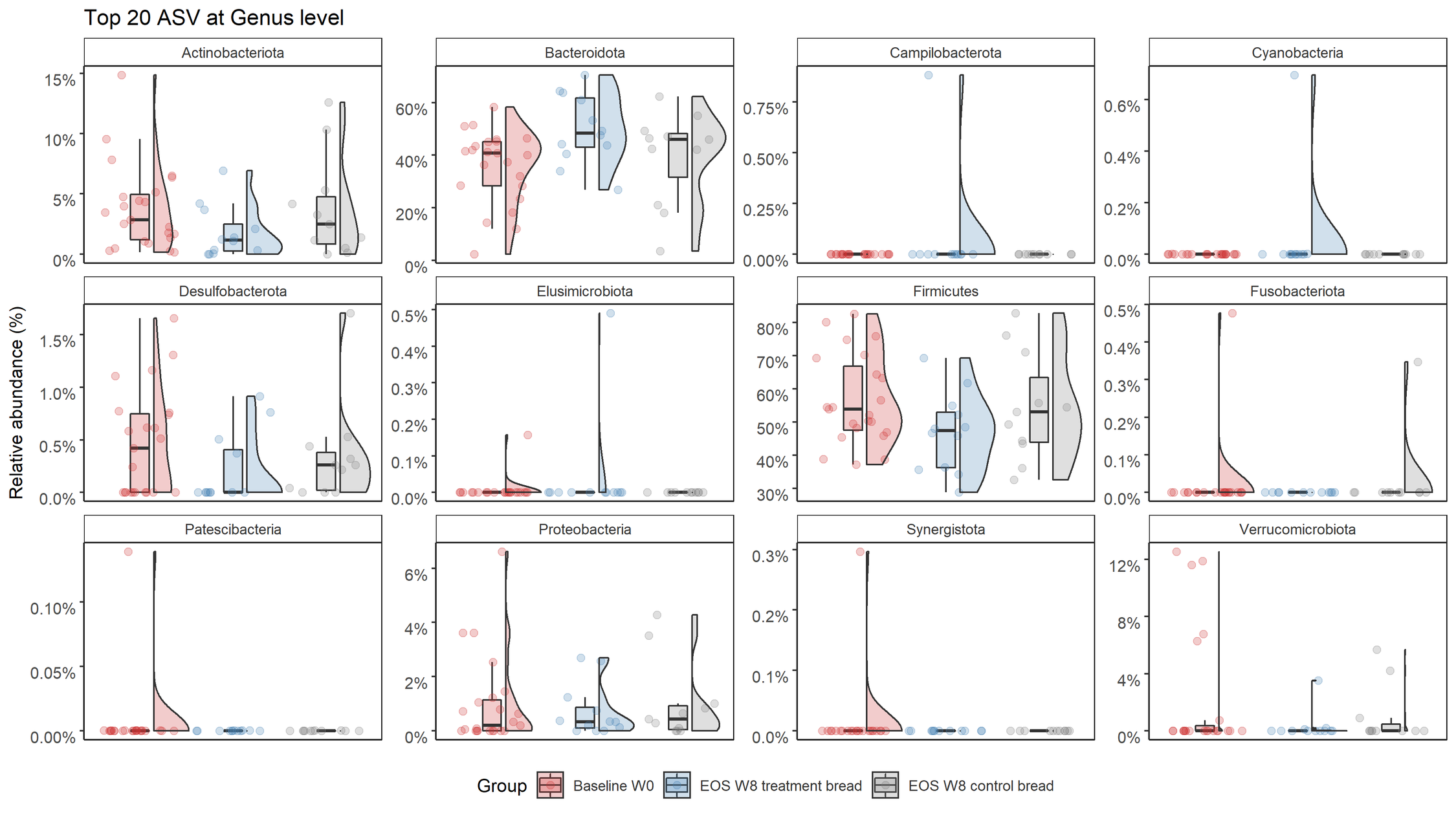

Supplement: S2 Fig — Samples were classified by group of treatment (Baseline w0, samples at baseline week 0; EOS w8 treatment bread, samples at the end of study visit week 8 after treatment bread; and EOS w8 control bread, samples at the end of study visit week 8 after control bread). (TIF) [file pone.0297836.s006.tif]

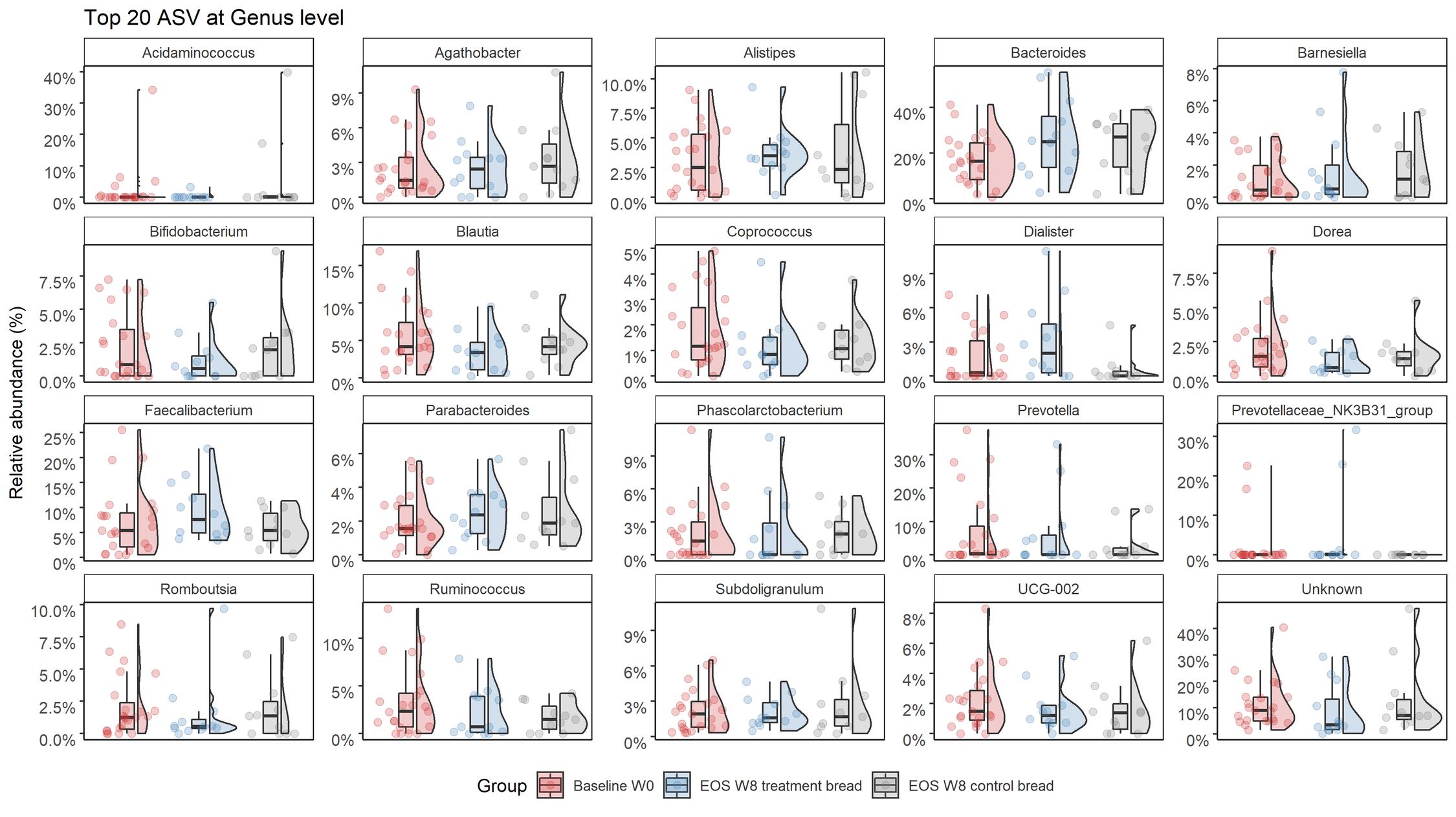

Supplement: S3 Fig — Samples were classified by group of treatment (Baseline w0, samples at baseline week 0; EOS w8 treatment bread, samples at the end of study visit week eight after treatment bread; and EOS w8 control bread, samples at the end of study visit week 8 after control bread). (TIF) [file pone.0297836.s007.tif]

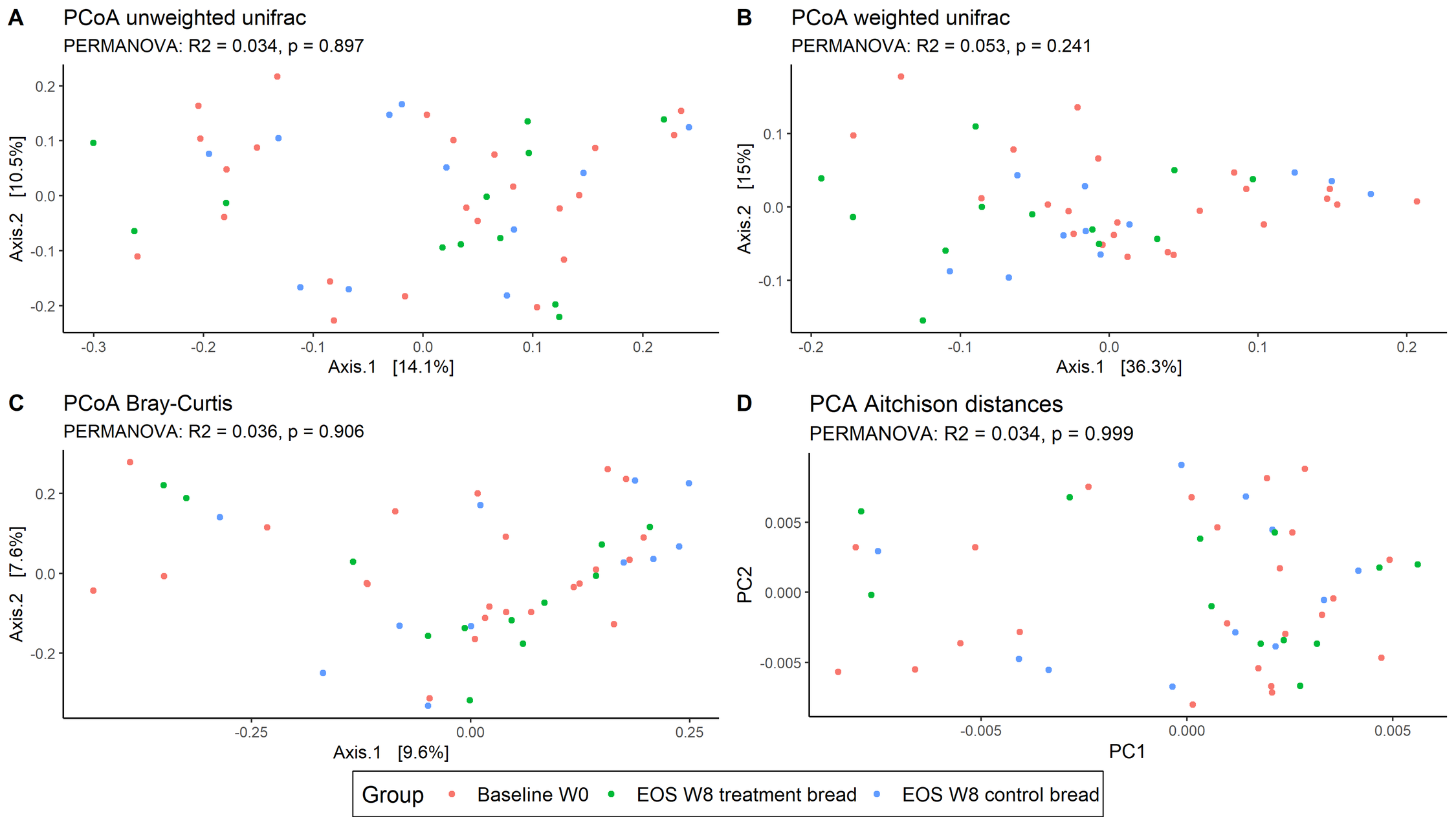

Supplement: S4 Fig — Principal coordinate analysis (PCoA) and Principal component analysis (PCA) with PERMANOVA tests of gut microbiota from the stool samples clustered in groups (Baseline w0, samples at baseline week 0; EOS w8 treatment bread, samples at the end of study visit week 8 after treatment bread; and EOS w8 control bread, samples at the end of study visit week 8 after control bread). Represented distances are based on unweighted (A) and weighted (B) UniFrac, Bray-Curtis dissimilarities (C), and Aitchison distances (D). (TIF) [file pone.0297836.s008.tif]
