# Supplementary material for: Impact of bread diet on intestinal dysbiosis and irritable bowel syndrome symptoms in quiescent ulcerative colitis: A pilot study
Source: PLoS One. 2024 Feb 16;19(2):e0297836. doi: 10.1371/journal.pone.0297836 (PMC10871487; doi:10.1371/journal.pone.0297836)
Supplement: S1 Protocol — (PDF) [file pone.0297836.s009.pdf]

Controlled, randomized and double-blind clinical trial to evaluate the effect *in vivo* of long-fermented Elias Boulanger bread (EBLF) compared to theshort-fermented industrial bread (CF) in patients with Ulcerative Colitis (UC) quiescent

Code of Protocol: RTC-2017-CU

Promoter: Consortium Retos - Collaboration: ELIAS Boulanger SL and group of intestinal diseases and microbiota of IDBGi.

Funding: Ministry of Economy, Industry and Competitiveness. Retos Program-collaboration 2017 RTC-2017-6467-2

Principal Investigator and Coordinator: Dr. Xavier Aldeguer Manté, Head of Service of Digestive, University Hospital of Girona, Dr. Josep Trueta and Santa Caterina Hospital, Salt

Centers where the study will be performed:

Hospital Universitari Dr. Josep Trueta, Avinguda de

França S/N, 17007, Girona, Spain.

Hospital Santa Caterina 17007, Salt, Girona, Spain

Centre where biological will be processed:

Group of research of intestinal disease and microbiota,

Institut de recerca de Biomedicina de Girona (IDIBGI),

Dr. Josep Trueta,Avinguda de França S/N, 17007, Girona, Spain

Duration: 36 months, from January 2019.

1. BACKGROUND

Numerous studies have now shown that the gut microbiota is a key factor in proper intestinal function but also in the regulation of the immune response in humans (1). Alterations in the intestinal microbiota are a determining factor in the development of serious chronic diseases. An example of this could be the case of Ulcerative Colitis (UC), where studies carried out with faecal or mucosa-associated microbial communities have shown that UC patients have an altered microbiota that differs from those individuals who do not have the disease (2). These alterations in the microbiota cause a constant antigenic stimulation, aggregated by genetic defects in the host, which will lead to a continuous activation of its immune system and lead to chronic intestinal damage (3). Thus, the intestinal microbiota is considered a direct marker of the state of intestinal inflammation (4).

Recent studies by our group have shown that patients with inflammatory bowel disease, such as UC, have a decrease in the diversity and bacterial proportions of their gut microbiota, specifically with a significant decrease in the abundance of *Faecalibacterium prausnitzii* and an increase in *Escherichia coli*. These changes in the composition of the gut microbiota are called dysbiosis and several studies have confirmed that these bacteria in particular are good indicators of dysbiosis in inflammatory bowel disease (2).

In an attempt to reverse this situation of dysbiosis, one possibility would be the use of prebiotics, which have the potential to stimulate the growth of beneficial intestinal bacteria in a selective manner (5); for example, *F. prausnitzii*, which is a butyrate producer and one of the three most abundant species in a healthy human gut (6). The importance of the abundance of butyrate- producing species in the intestinal microbiota is reflected in the anti-inflammatory activity of this compost (7).

Among the wide variety of prebiotic foods, in this case we are interested in a common food in the Mediterranean diet, so as not to excessively change the diet of the sick. Pa is a potentially prebiotic food with a high percentage of insoluble dietary fibre. Dietary fibre induces the production of short-chain fatty acids, mainly acetic, propionic and butyric, which are important nutrients for epithelial cells and intestinal microbiota. It therefore has the capacity to promote the growth of butyrate-producing bacteria in the intestinal microbiota (3). A high quality, long-fermenting, compact tidal mass could have beneficial prebiotic effects both for the general population and for those patients with UC in remission, but who continue to show symptoms compatible with irritable bowel syndrome. If t h e prebiotic effect of these pills is confirmed, they could even be recommended by health workers to the general population as a prevention or alleviation of symptomatology caused by intestinal inflammation.

Thus, from the perspective of public health and as a personal interest of our group in collaboration with ELÍAS FORNER S.L., we believe that it would be interesting to know more about the impact of bread on the microbiota-inflammation relationship of the intestinal mucosa; and whether the intake of traditionally made bread, with a lot of salt and long fermentation, could be an advantage for our study population.

2. HYPOTHESIS AND OBJECTIVES

2.1. Hypothesis

Eating bread made from stone-ground wheat flour and compact dough with prolonged fermentation processes is beneficial for people with mild Ulcerative Colitis (UC), but who have symptomatology compatible with Irritable Bowel Syndrome (IBS), through the modulation of their intestinal microbiota.

2.2 Objectives

To compare *in vivo* the influence of two types of bread, one long fermented and one short fermented, on the intestinal microbiota of patients with cystic Ulcerative Colitis (UC) with symptomatology compatible with IBS (Rome IV criteria).

Specific objectives:

- To determine the abundance of *Faecalibacterium prausnitzii/Lactobacillus/Escherichia coli* in patients with quiescent UC, before (V_0) and after 8 weeks (V_F) of ingestion of long-fermented or short-fermented bread.
- To determine the correlation between the changes observed in the composition of the intestinal microbiota at V_0 and V_F and the clinical manifestations of the patient with cystic fibrosis.
- To assess the impact of long-fermenting versus short-fermenting bread on improving the clinical response of patients with cystic fibrosis.
- To determine the microbiological profile of the microbiota of UC patients before (V_0) and after the intervention (V_F) of eating bread by means of mass sequencing.

3. MATERIAL AND METHODS

3.1. Type of study/design

Prospective, randomised, double blind study.

3.2. Population of study

The proposed study will include 96 patients from the Digestive Department of the University Hospital of Girona Dr. Josep Trueta and Hospital Santa Caterina de Salt. The recruitment will be coordinated by Dr. Xavier Aldeguer and Dr. Anna Bahí.

They will be patients diagnosed with UC, who at the time of inclusion in the study are in remission (Mayo ≤2), but who present symptomatology compatible with Irritable Bowel Syndrome (IBS). This symptomatology is present in 35% of patients with UC in remission.

3.3. Inclusion criteria

- > 18 years old with established diagnosis of UC in remission/non-active phase (Mayo Index ≤2 and Calprotectin ≤ 250ug/gr)
- Completion of Rome Criteria IV

- Symptomatology compatible with Irritable Bowel Syndrome (Moderate-green severity index IBS-SSS >175)
- Informed consent signed

3.4. Exclusion criteria

- Active Ulcerative Colitis (Mayo Index ≥2 , Calprotectin ≥ 250 ug/gr)
- Antibiotic treatment 3 months prior to the inclusion of the study
- Probiotics and/or prebiotics for the previous 3 months
- Continued use of non-steroidal anti-inflammatory drugs (NSAIDs)
- Coeliac Disease
- Colectomy or other surgeries (intestinal resections) compromising the intestinal tract
- Pregnancy or breastfeeding
- Alcoholism or active drug dependence
- Incapacity to give consent

3.5. Sample management

The samples will be taken by the patient to the Gastroenterology, Hepatology and EndoscopyService of the University Hospital of Girona Dr. Josep Trueta or to the Gastroenterology Service of the Santa Caterina Hospital in Salt. The sample must be preserved and transported, as far as possible, in a cool place in a portable refrigerator or gel plate. These samples will be processed by the Digestion Research Group of the IdIBGi. Finally, the leftover samples will be destroyed or stored at the Hospital's Biobank according to the patient's consent.
The blood samples will be taken directly from the Hospital Universitari de Girona Dr. Josep Trueta or the Hospital Santa Caterina de Salt. They will be analysed at the clinical analysis laboratory of the Hospital de Girona Dr. Josep Trueta and the results will be communicated to the corresponding doctor using the electronic medical records platform "SAP". Leftover blood samples will be destroyed.

3.6. Interventions

The intervention of the study will consist of the daily consumption of 150-200g of one of the two types of bread under study, a long fermentation bread (EBLF) and a short fermentation bread (CF), for 8 weeks by the participants.

Long-fermented bread (EBLF) has the following composition: wholemeal flour (ground on the stone), water, freeze-dried Paris milk (<1%), compact dough and salt. The fermentation is of the aceto-lactic type for more than 40 hours.
On the other hand, the short fermentation pa (CF) has the following ingredients: refined flour, water, salt and milk. With a rapid fermentation totally controlled in a maximum of 2 hours.
Elias Forner will provide the necessary bread for the daily consumption of the participants at the centres, where the patients will collect it 1 or 2 times a week during the 8 weeks of the intervention.
All the breads will be prepared with the same presentation by Elias Forner. All the members of the research team will be responsible for assigning the intervention.
No dietary or lifestyle interventions (diet, physical activity, etc.) will be carried out in either of the two

experimental groups.

As a mechanism to control the consumption of bread during the study, and in order to guarantee internal validity, each participant will be provided with a calendar where they will have to write down each day whether or not they have consumed the 150-200g of bread per day referred to in the study (Annex 1). During the 8 weeks of the study, participants will be reminded that they have to fill in the calendar and that, after the 8 weeks, the calendar will be returned to the IdIBGi researchers. This information will be contrasted with ELÍAS Forner S.L. and with the patient during the on-site visit.

3.7. Randomization

Participants will be assigned to one of the two interventions using a randomised sequence separated by sex. The double blindness will be maintained throughout the study. In the case of withdrawal or abandonment of a participant, the new subject will be assigned a consecutive number and the intervention will be assigned using the same randomised sequence separated by sex as the rest of the participants.

3.8. Variables

a) Variables demographics

- **Age:** expressed in years (a)
- **Gender:** male (M) / female (F)
- **Weight:** expressed in BMI
- **Ethnicity:** Caucasian / African / Asian / american
- **Tobacco:** Yes/ No/ Ex-smoker
- **Family history:** If not. Which one type of disease?

b) <u>Clinical variables:</u>

- **Mayo Clinical** (*Clinical colitis activity index*) (Schroeder KW *you at the. NEngl J Med* 1987; 317: 1625-9). Range of score: 0-9 points

- **Rome IV criteria** (Lacy BE, Mearin F, Chang L, *et al.* Bowel disorders. Gastroenterology 2016;150:1393-407). IBS is diagnosed by the presence of recurrent abdominal pain that must be present at least one day per week, with two or more of the following characteristics: a) it is associated with defecation; b) it is related to a change in stool frequency; and c) it is related to a change in stool consistency. As for the duration of discomfort requirements, it should be taken into account that the criteria must have been met during the last three months and the symptoms must have started at least six months prior to diagnosis.

- **IBS- SSS** (*Irritable Bowel Syndrome - Symptom Severity Score*) (Almansa C *et al*. Rev Esp Enferm Dig 2011 Dec;103 (12):612-8. *Score* validated in Spanish, to evaluate: 1- the intensity of pain, 2- the frequency of pain, 3- the intensity of

abdominal distension, 4- satisfaction with the stool habit and 5- the impact on the patient's functioning in daily life, which evaluates each item from 0 to 100.

- **CVE 20** (Quality of Life Questionnaire for Constipation Patients) (Perona, M. *et al* Med Clin (Barc). 2008 Sep 27;131(10):371-7). A quality of life questionnaire validated in Spanish which consists of 20 items and 4 dimensions (Emotional (10), Physical/general(8+1), Rectal(3), Social(3)).

- **HADS** (*Hospital Anxiety and Depression Scale*) (Johnston M, *et al*. J Psychosom Res 2000;48:579-84.9) consists of two sets of seven questions - one representing the anxiety subscale and the other the depression subscale - with the two psychopathological concepts of anxiety and depression being independent. Each item is rated according to a four-point frequency scale ranging from 0 to 3.
- **Likert scale:** To assess the response of "subjective" improvement of gastrointestinal symptoms after the intervention, by means of the categorical question: Your IBS symptoms are 1- Much worse, 2- Worse, 3- The same, 4- Better and 5- Much better.

- **Mediterranean diet adherence questionnaire (**Estruch R et al NEJM 2013). Consists of a set of 14 short questions.

c) **Analytical parameters:**

Hemoglobin: hemoglobin concentration in blood (g/dl).
Albumin: albumin concentration in blood (g/dl).

Triglycerides: triglyceride concentration in blood (mg/dl).

Cholesterol: total cholesterol concentration in blood (mg/dl).
C-reactive protein: concentration of C-reactive protein in blood (mg/dl).

Fecal calprotectin: concentration of calprotectin in blood (µg/g).
Ionogram: plasma ion concentration.

Sodium: concentration of sodium in blood (Na, mEq/l).
Potassium: blood potassium concentration (K, mEq/l).
Calcium: concentration of calcium in blood (Ca, mEq/l).
Phosphorus: concentration of phosphorus in blood (P, mEq/l).
Chlorine: concentration of chlorine in blood (Cl, mEq/l).
Iron: concentration of iron in blood (Faith, mEq/l).

d) Microbiological variables

The abundance of *Lactobacillus*, *Faecalibacterium prausnitzii* and *Escherichia coli* (AIEC: Adherent - Invasive E. coli) in the females of the participants included in the study will be analysed (Martinez-Medina et al., 2006; Martinez-Medina et al., 2009; Lopez-Siles et al., 2014).
The microbiological profile of the microbiota of these patients will be analysed by means of mass sequencing, with the subcontracting of the company StarSEQ.

3.9. Methods

Included are 96 patients with UC in remission (Mayo Index ≤2 and Calprotectin ≤ 250ug/g), over 18 years of age, who have signed the informed consent form, meet the Rome IV criteria and have a moderate to severe severity index (IBS-SSS >175).

These patients will be randomised to one of the two intervention groups (N=48 per group):

- Intervention A: Bread EBLF (150-200g daily).
- Intervention B: Bread CF (150-200g daily).

For both intervention groups, A and B, stool and blood samples will be collected before starting the intervention (Visit 0 - Baseline), and after 8 weeks (Final Visit). The monitoring period will be 8 weeks. In addition, demographic data (age, sex, tabacco habit, etc.), clinical data (Mayo Index, **Rome IV**, **IBS- SSS, CVE 20, HADS, Likert Scale** PCR, ESR, albumin, haemoglobin, faecal calprotectin, etc.), and microbiological variables will also be collected at V_0 and V_F.

The intervention will last 8 weeks and the following visits will take place during the study:

1. Consent/Pre-Randomization Visit

The patient will be assessed by a doctor from the Gastroenterology Department of the Hospital Universitari Dr. Josep Trueta who will explain the study and assess the inclusion and exclusion criteria. Participants will be able to ask any questions they may have before signing the written consent form. If the potential volunteer wishes to participate, after reading the informative documentation and receiving the relevant explanations, he/she must read the informed consent form carefully and sign it if he/she agrees with it.
The patient will be provided with the 2 containers necessary to collect the sample at home.

2. Baseline Visit (V_0)

The patient will come to the visit with the sample of the femina corresponding to the baseline visit (V_0) prior to the intervention.

Demographic variables will be collected (age, sex, weight, ethnicity, smoking habits, family history, etc.), the different established questionnaires and indices (Rome IV, Mayo, IBS-SSS, CVE-20, HADS) will be carried out, and a blood test (PCR, Calprotectin, haemogram, etc.) will be performed.

The patient will be randomised and will be provided with bread according to the result of randomisation to intervention A or B. The patient will be told that they can collect the following

portions of bread at the centre 1 or 2 times a week for the next 8 weeks of the intervention.

3. End of Study Visit (V_F)

The patient will come to the visit, 8 weeks later, with the sample of the female body corresponding to the final visit (V_F) of the study.

The different established questionnaires and indices (Rome IV, Mayo, IBS-SSS, CVE-20, HADS, Likert scale) and a blood test (PCR, Calprotectin, haemogram, etc.) will be carried out.

The evolution of the patient's symptomatology will be evaluated 8 weeks after the operation, and a new medical evaluation will be carried out.

3.10. Microbiological analyses

Fecal samples collected from V_0 and V_F will be incubated at -80ºC at the Institute for Biomedical Research of Girona (IdIBGi). Before the microbiological analysis, DNA extraction of the 16s RNA gene will be performed using the commercial NucleoSpin® Soil Kit (Machery-Nagel GmbH & Co., Germany). The DNA concentration will be determined with the Qubit® BR kit (Invitrogen). The genomic DNA will be amplified by qPCR using the TaqMan probe system (Applied Biosystems). Specific primers will be used for the quantification of *Escherichia coli, Faecalibacterium prausnitzii,* Eubacteris and *Lactobacillus*. All quantitations will be performed in duplicate using the Stratagene Mx3005P thermal cycler. Internal amplification controls are included.

The microbiological profile of the microbiota of these patients will be analysed by mass sequencing by the subcontracted company StarSEQ. The region corresponding to the V3-V4 variable region of the 16S rRNA gene will be sequenced using specific primers for Illumina HiSeq200 technology through *paired-end reads* (generating sequences of 300bp).

3.11. Sample size

We used the main variable, concentration of *F.* praunitzi/lactobacillus/E*.coli*, to calculate the sample size. Given the absence of normative data on the concentration of these microbial populations in the intestinal microbiota, a sample size has been estimated according to the magnitude of the effect or measure of the difference between the two groups (Bread EBLF and bread CF).

Specifically, we decided to look for a sample measure that would allow us to observe theoretically a *size* effect difference of 0.6 in the composition of the microbiota of the two groups, according to the criteria described by Cohen (8).

Student's t-test will be applied to 2 independent, normally distributed samples. The standard deviations of the 2 groups are unknown and will be estimated based on the data obtained in the study. We assume a significance level of α=0.05, bilateral contrast and a power of the study of 80% (β=0.02). Software G*Power Version 3.1.9.2 will be used; Module: *Means difference Between two independent means (two groups)*.

The result is that a sample of 80 patients (40 per group) allows us to find a difference of 0.6 in the concentration of the target microorganisms in both populations. Thus, and foreseeing a 15% loss of follow-up, 96 patients will be randomised to ensure the final entry of the 80 patients needed according

to the sample calculation above.

3.12. Statistical analyses

A statistical analysis of variance (ANOVA) with repeated measures and a Friedman test (for parametric and non-parametric variables, respectively) will be applied to compare bacterial abundance at different times of the intervention (V_0 and V_F) and to compare between intervention groups A and B. Statistical significance will be taken into account when $p \leq 0.05$.

A multiple linear regression model will be fitted to determine the correlation between microbiological abundance values (*F. prausnitzii / E. coli*) in comparison with the co-variables. The clinical variables will be: inflammation markers such as Mayo, faecal calprotectin, CRP, haemoglobin, albumin, etc.

As a secondary variable of analysis in the study on the impact of Bread EBLF vs Bread CF on clinical IBS, the clinical response after 8 weeks will be used, defined as a subjective improvement on a Likert scale of "Subjective response rating" as better or much better than the baseline situation and accompanied by a decrease in the IBS-SSS score greater than 30% of baseline or reaches a score below 75 points. ANCOVA will be used to evaluate the effect of the intervention and the following covariates w i l l b e included: baseline IBS-SSS, HADS score, sex, BMI.

All statistical procedures will be performed with SPSS 15.0 [IBM, Chicago, IL, USA].

4. ETHICAL ASPECTS

The research will respect the fundamental principles of the Helsinki Declaration, the Council of Europe Convention on Human Rights and Biomedicine, as well as the requirements established by Spanish legislation in the field of biomedical research, the protection of personal data and bioethics complying at all times with the general data protection regulation (No. 2016/679) of the European Parliament and of the Council of 27 April 2016.

5. CHRONOGRAPH

	2019												2020												2021											
**Duties**	G	F	M	A	M	J	J	A	S	O	N	d	G	F	M	A	M	J	J	A	S	O	N	d	G	F	M	A	M	J	J	A	S	O	N	d
CEIC approval	■																																			
Recruitment		■	■	■	■	■	■	■	■	■	■	■	■	■	■	■	■	■	■	■	■	■	■	■	■	■	■	■	■	■	■	■				
DNA extraction and Quantification			■	■	■	■	■	■	■	■	■	■	■	■	■	■	■	■	■	■	■	■	■	■	■	■	■	■	■	■	■	■	■			
qPCR of the feces													■	■	■	■	■	■	■	■	■	■	■	■	■	■	■	■	■	■	■	■	■	■	■	
16S sequencing													■	■	■	■	■	■	■	■	■	■	■	■	■	■	■	■	■	■	■	■	■	■	■	
Analyzes of the generated data generated and final report																		■	■	■	■	■	■	■	■	■	■	■	■	■	■	■	■	■	■	■

6. BUDGET

This study is funded through the grant Retos - Col-laboració RTC-2017-6467-2 from the Ministry of Economy, Industry and Competitiveness. The budget of the Digestive Research Group allocated to the clinical trial is €32,300.

ELÍAS FORNER SL, as promoter of the study and with the Challenges-Collaboration Grant obtained, will assume the expenses generated by the supply of the bread and the expenses incurred by the patients (food and travel) how as a result to be part of the study.

7. BIBLIOGRAPHY

1. Geuking MB, Köller Y, Rupp S, McCoy KD. The interplay between the gut microbiota and the immune system. Gut Microbes 2014 May-Jun;5(3):411-8.

2. Lopez-Siles M, Martinez-Medina M, Busquets D, Sabat-Mir M, Duncan SH, Flint HJ, Aldeguer X, Garcia-Gil LJ. Mucosa-associated *Faecaelibacterium prausnitzii* and *Escherichia coli* co- abundance dog distinguish Irritable Bowel syndrome and Iflammatory Bowel Disease pphenotypes. Int J Med Microbiol. 2014 May;304(3-4):464-75.

3. Balfour tailor R. reviews in basic and clinical gastroenterology Gastroenterology. 2008;134:577-594.

4. Walker AW, Sanderson JD, Churcher C, Parkes GC, Hudspith BN, Rayment N, Brostoff J, Parkhill J, dougan G, Petrovska L. High-throughput clone library analysis of the mucosa- associated microbiota reveals dysbiosis and differences between inflamed and non-inflamed regions of the intestine in inflammatory bowel disease. BCM Microbiology. 2011 Jan; 11:7.

5. Patel A, Herbert L. News approaches for bacteriotherapy: prebiotics, new-generation probiotics, etc synbiotics CID. 2015:60.

6. Eckburg PB, Bik EM, Bernstein CN, Purdom E, Dethlefsen L, Sargent M, Gill SR, Nelson KE, Relman yes Diversity of the human intestinal microbial flora Science. 2005 Jun;308(5728):1635- 1638.

7. Gonçalves P, Araújo JR, Di Santo JP. A cross-talk between microbiota-derived short-chain fatty acids and the host mucosal immune system regulated intestinal homeostasis and inflammatorybowel disease. Inflamm Bowel Dis. 2018 Feb;24(3):558-572.

8. Cohen, J. Statistical power analysis for the behavioral sciences. 1988, 2nd ed. Hillsdale, NJ: Lawrence Erlbaum Associates.

9. Faul, F., Erdfelder, E., Lang, A.-G., & Buchner, A. G*Power 3: A flexible statistical power analysis program for the social, behavioral, and biomedical sciences. Behavior Research Methods. 2007(39):175-191.

10. Faul, F., Erdfelder, E., Buchner, A., & Lang, A.-G. Statistical power analyzes using G*Power 3.1:
