# Supplementary material for: Impact of bread diet on intestinal dysbiosis and irritable bowel syndrome symptoms in quiescent ulcerative colitis: A pilot study
Source: PLoS One. 2024 Feb 16;19(2):e0297836. doi: 10.1371/journal.pone.0297836 (PMC10871487; doi:10.1371/journal.pone.0297836)
Supplement: Attachment — Submitted filename: Response to Reviewers.docx [file pone.0297836.s011.docx]

**Response to the editor/reviewers-** “**Impact of bread diet on intestinal dysbiosis and irritable bowel syndrome symptoms in quiescent ulcerative colitis: A pilot study” (PONE-D-23-21156)**

We thank the editor for their positive response and consideration of our manuscript for publication. We fully addressed all the points raised by the academic editor and reviewers on a point-by-point basis. Please, find below our responses:

**Academic editor:**

*“Please review your reference list to ensure that it is complete and correct. If you have cited papers that have been retracted, please include the rationale for doing so in the manuscript text, or remove these references and replace them with relevant current references. Any changes to the reference list should be mentioned in the rebuttal letter that accompanies your revised manuscript. If you need to cite a retracted article, indicate the article’s retracted status in the References list and also include a citation and full reference for the retraction notice”*

**Response:** We have carefully reviewed the reference list and ensured that it is complete and correct.

**Reviewer 1:**

*“The modifications made based on the review as well as questions made have been answered with great effort - this is highly appreciated. It is clearly visible that the manuscript has greatly improved and its scientific quality and reliability as well as clarity for the readers have markedly been increased.”*

**Response**: We appreciate the Reviewer 1 comments and agree with the improvement of the scientific quality of our manuscript.

**Reviewer 3:**

*Minor revisions:*

1- *“Line 119: Grammatical correction: Furthermore, subjects were asked not to alter their diet during the intervention period.”*

**Response**: We thank Reviewer 3 for their valuable comments and for revising the manuscript. We have corrected the grammatical error as suggested.

2- *“Line 163: Remove "during December 2019 and August 2021". The sentence that follows provides more clarity.”*

**Response**: We have removed the sentence as suggested.

3- *“Table 1: Identify the statistical testing method(s) used to estimate the p-values in Table 1. To improve clarity, consider moving Table 1 after the "Statistical analysis of blood test and questionnaire data" section. This section seems to include the statistical testing methods used to estimate p-values in Table 1.”*

**Response**: We have specified the statistical test used in Table 1 to estimate the p-values (lines 213-215). While we acknowledge the potential enhancement in clarity by relocating Table 1 after the statistical analysis section, we have positioned it immediately after its first mention following requirements stipulated by the journal.

4- *“Line 220: Indicate the statistical testing method which achieves 80% power. Perhaps it is the t-test.”*

**Response**: We have included the statistical test used in the revised manuscript (line 221 of the revised manuscript).

5- *“Line 231: Indicate the underlying covariance structure used in the Linear Mixed Models and the criteria for selecting it.”*

**Response**: In our Linear Mixed Models (LMM) for analyzing the effects the intervention, we incorporated a random intercept term with a variance components structure to capture subject-specific variability. This choice facilitated the modeling of subject-specific variability while maintaining a straightforward and interpretable model. Model fit diagnostics supported the appropriateness of the selected covariance structure. We have specified the covariance structure used in the manuscript (line 235).

6- *“Tables 2 and 3: A) In addition to the frequencies, provide percentages that correspond to them. B) State the sample sizes of the groups in the header row.”*

**Response**: We have included the percentages and sample sizes in Tables 2 and 3 as suggested.
