# Supplementary material for: Impact of bread diet on intestinal dysbiosis and irritable bowel syndrome symptoms in quiescent ulcerative colitis: A pilot study
Source: PLoS One. 2024 Feb 16;19(2):e0297836. doi: 10.1371/journal.pone.0297836 (PMC10871487; doi:10.1371/journal.pone.0297836)
Supplement: Attachment — Submitted filename: Response to Reviewers.docx [file pone.0297836.s012.docx]

**Response to the editor/reviewers-** “**Impact of bread diet on intestinal dysbiosis and irritable bowel syndrome symptoms in quiescent ulcerative colitis: A pilot study” (PONE-D-23-21156)**

We thank the editor for their positive response and consideration of our manuscript for publication. We fully addressed all the points raised by the academic editor and reviewers on a point-by-point basis. Please, find below our responses:

**Academic editor:**

*“Please review your reference list to ensure that it is complete and correct. If you have cited papers that have been retracted, please include the rationale for doing so in the manuscript text, or remove these references and replace them with relevant current references. Any changes to the reference list should be mentioned in the rebuttal letter that accompanies your revised manuscript. If you need to cite a retracted article, indicate the article’s retracted status in the References list and also include a citation and full reference for the retraction notice”*

**Response:** We have carefully reviewed the reference list and ensured that it is complete and correct.

**Reviewer 3:**

*Minor revisions:*

1- *Table 1: If data is normally distributed, summarize using means and standard deviations. If data is non-normally distributed, summarize using medians, first and third quartiles.*

**Response**: We thank Reviewer 3 for their valuable comments and for revising the manuscript. We have modified Table 1 as suggested.

2- *Tables 2 & 3: Consider displaying each level for categorical factors in separate rows rather than by separating with backslashes. This is the typical style for displaying categorical data in tabular form.*

**Response**: We appreciate Reviewer’s valuable comment regarding Table 2 and 3 style. We have modified categorical factors to display each level as a separate row as suggested. Furthermore, table footnote has also been modified accordingly.
